# The Effects of *Lactobacillus farciminis* and *Lactobacillus rhamnosus* on Growth, Blood Biochemical, and Meat Quality Indicators of Specific Pathogen-Free Broiler Chickens

**DOI:** 10.1155/2023/6297068

**Published:** 2023-07-04

**Authors:** Sabine Eglite, Aija Ilgaza, Lauma Mancevica, Maksims Zolovs

**Affiliations:** ^1^Preclinical Institute, Faculty of Veterinary Medicine, Latvia University of Life Sciences and Technologies, K. Helmana Street 8, Jelgava, LV 3004, Latvia; ^2^Statistics Unit, Riga Stradins University, Balozu Street 14, Riga, LV 1007, Latvia; ^3^Department of Biosystematics, Institute of Life Sciences and Technology, Daugavpils University, Parades Street 1a, Daugavpils, LV 5401, Latvia

## Abstract

The aim of our study was to evaluate the effects of *Lactobacillus farciminis* and *Lactobacillus rhamnosus* on live weight gain, feed consumption indicators, and some metabolic blood biochemical and meat quality indicators of specific pathogen-free Ross 308 broiler chickens. We carried out the study in three trials and included a total of 780 unsexed Ross 308 chickens, which we randomly divided into two groups: the control group (Con, *n* = 390, basal diet) and the probiotic group (ProL, *n* = 390, basal diet + a powder consisting of *L. farciminis* and *L. rhamnosus* 4 g/10 kg of feed). We raised broilers until day 35. We determined the amount of feed consumed, the average daily weight gain, the feed conversion ratio, the average daily feed intake, and the cumulative feed intake once a week. We collected blood samples from 45 broilers from each group at the end of the study. In addition, we slaughtered 30 broilers from each group by cervical dislocation to obtain a breast muscle sample (without skin) to determine meat quality in these chickens (cholesterol and unsaturated, omega-3, omega-6, omega-9, and saturated fatty acids). Feeding a probiotic mixture containing *L. farciminis* and *L. rhamnosus* did not significantly affect the growth and feed intake indicators. Feeding these probiotics significantly lowered the blood serum cholesterol levels but did not provide the expected reduction in meat cholesterol levels. However, feeding a probiotic mixture increased the levels of polyunsaturated fatty acids (omega-3 and omega-6 fatty acids) in the breast meat and decreased saturated fatty acids. To better explain the effect of the combination of lactic acid bacteria (*L. farciminis* and *L. rhamnosus*) on the growth and development of broiler chickens in our study, histological and immunohistochemical examinations should be performed.

## 1. Introduction

Probiotics are living microorganisms that, when administered in adequate amounts, improve the health of the host. Other definitions have been developed over the years that specify their mechanisms of action, site, delivery format, method, or host [1]. The positive effects of probiotics on the body have been widely studied in both human and veterinary medicine. The use of probiotics in farm animals has become an important research topic since 2006, when the use of antibiotics for disease prevention and productivity stimulation was banned in the European Union [2]. Poultry industry producers face various problems such as a reduced growth rate, dysbacteriosis of the digestive tract, and enteritis caused by various pathogens [3, 4]. Solutions to these and other problems are still being sought, and research has been conducted to prove the positive effects of probiotics on the host.

The effects of probiotics on growth performance have been widely studied. For example, Hussein and Selim [5] reported that feeding multistrain probiotics to broilers significantly reduced daily feed intake and improved feed conversion compared with the control group. Similarly, Chen et al. [6] fed *Lactobacillus rhamnosus* to broilers (white leghorns) and observed a significantly increased live weight and average daily gain as well as a significantly decreased feed conversion ratio (FCR) at the end of the study. However, Qorbanpour et al. [7] fed male Ross 308 broilers multistrain probiotics (including *Lactobacillus acidophilus* and *Lactobacillus casei*) and showed that the experimental diet had no effect on daily feed intake, weight gain, and overall feed conversion throughout the study period. Zhu et al. [8] obtained similar results: feeding female broilers probiotics consisting of thermally inactivated *Bacillus subtilis* and *L. acidophilus* in a 1 : 1 ratio, the authors reported a significantly reduced FCR but found no significant differences in parameters such as final body weight, average daily gain, and average daily feed intake between groups. These contradictory results indicate that additional studies in this field are necessary.

The impacts of probiotics on biochemical indicators of protein and fat turnover in the blood of birds are still subject to debate. In several studies, broilers fed probiotics containing various *Lactobacillus* spp. presented significantly higher amounts of total protein, including albumin and globulin, as well as significantly lower concentrations of cholesterol, total lipids, and triglycerides in the blood [5, 9, 10]. However, in several studies, when broilers were fed probiotics that included *Lactobacillus* spp., there were no significant changes in the biochemical blood parameters at the end of the study [7, 8, 11].

Currently, consumers demand cheap but high-quality and nutrient-rich food. Therefore, producers and researchers have focused on improving not only feed conversion and bird productivity, but also the quality of the obtained products, including poultry meat. According to previous studies, when broilers are fed probiotics, the water-binding capacity of the meat is increased [4, 12], along with the percentages of moisture, protein, and ash in meat. In contrast, the fat content is reduced [13, 14], along with the cholesterol content [13, 15]. In poultry meat, the total amounts of saturated fatty acids (SFAs) and unsaturated fatty acids and their ratio are important quality indicators. Poultry meat contains long-chain polyunsaturated fatty acids (LC-PUFAs) and is a source of omega-3 (*ω*-3), omega-6 (*ω*-6), and omega-9 (*ω*-9) fatty acids. Various studies have shown that probiotic feeding can increase the levels of PUFAs and reduce the levels of SFAs in poultry meat, thereby affecting the ratios of LC-PUFAs and SFAs [16, 17]. Alagawany [18] reviewed the results regarding various sources of *ω*-3 and *ω*-6 fatty acids and concluded that producers and scientists, using various vegetable oils or products of fish origin as feed additives, often face the problem of oxidative stability of the obtained poultry meat. LC-PUFAs in meat are highly susceptible to oxidation, resulting in off-flavours and off-odours in poultry meat, which negatively affect meat quality and consumer acceptability. The authors acknowledged that it is necessary to continue looking for ways to increase the amount of *ω*-3 fatty acids in poultry meat and to bring the *ω*-6/*ω*-3 ratio closer to 1–4/1 [18].

Our research has shown that a feed supplement containing *L. farciminis* and *L. rhamnosus* activates the internal reserves of the alimentary canal of broiler chickens and facilitates the digestion of industrially produced feed. This would allow increasing the quantitative and qualitative indicators of the meat, without harming or perhaps even improving the health status of fast-growing broilers [19]. The aim of our study was to determine the effects of *L. farciminis* and *L. rhamnosus* on live weight gain, feed consumption indicators, and some metabolic blood biochemical and meat quality indicators of specific pathogen-free Ross 308 broilers.

## 2. Materials and Methods

### 2.1. Ethical Approval

All issues related to the keeping of birds were regulated by the Republic of Latvia Cabinet Regulation No. 98, adopted on 2 February 2010, “Welfare Requirements for Keeping and Use of Chicken for Meat Production” [20]. The study was approved by the Research Committee of the Faculty of Veterinary Medicine, Latvia University of Life Sciences and Technologies (protocol No.2021/1).

### 2.2. Experimental Design and Animal Management

The experimental part of the study was conducted at the Clinical Research Center, Faculty of Veterinary Medicine, Latvia University of Life Sciences and Technologies, Jelgava, Latvia, from April to December 2021. The study was organised in three trials, namely, from April 21 to May 26, from June 22 to July 27, and from November 10 to December 14. All trials were identical, and the results for each relevant group were combined and analysed. In total, 780 (260 in each trial) specific pathogen-free Ross 308 broilers (males and females) were obtained after hatching from the commercial hatchery “Kekava” (chicks were not infested with any specific pathogens). The broilers were weighed and randomly divided into two groups: the control group (Con, *n* = 390 (130 in each trial)) and the probiotic group (ProL, *n* = 390 (130 in each trial)). The groups were placed in two identical rooms (biochambers) with full microclimate control (temperature, humidity and air supply, control of incoming and outgoing air composition, and light mode) and video surveillance. The volume of each room was 9 m^3^. The birds were placed on deep bedding, using clean pine and spruce shavings as bedding material. In both groups, during the first week, the ambient temperature in the chambers was maintained at 32–34°C, which, as the birds grew, was gradually reduced to reach 20°C by the end of the study. The daily light regime on the first day was 23 h of light and 1 h of darkness (23 h/1 h). After that, the dark regime was gradually extended, and from days 7 to 26, the light regime was 18 h light and 6 h dark (18 h/6 h). In the last week of the study, the dark regime was gradually reduced until it reached 20 h of light and 4 h of dark (20 h/4 h).

### 2.3. Diet and Supplementation of *Lactobacillus* spp

Fresh drinking water was provided *ad libitum* in the drinking lines. The basal diet for both groups (Con and ProL) was the same and was composed according to the age of the chicks: starter (from day 0 to 10), grower (from day 11 to 24), and finisher (from day 25 until the end of the study). The analytical composition and physiological additives of the diet at each stage of feeding are shown in Table 1. The ration was prepared based on the Ross 308 breeding guidelines [21]. The starter diet contained wheat grains, soy sprouts, vegetable oil, corn gluten, monocalcium phosphate, fish meal, calcium carbonate, sodium chloride, and sodium sulphate. The grower and the finisher diets contained no fish meal, but they did contain rapeseed and fatty acids.

We added a powder containing lactic acid bacteria to the feed of the ProL group, consisting of *L. farciminis* (CNCM-I-3699-7.8.106 GU/g) and *L. rhamnosus* (CNCM-I-3698-7.8.106 GU/g), at estimated doses of 4 g per 10 kg of feed (initially mixing the probiotic powder into 1 kg of feed, then mixing it with the rest of the daily feed ration for 5 minutes), according to the conditions provided by the manufacturer (STI Biotechnologie, France) [22]. The mixture was stored and used exactly as recommended by the scientists involved in the production.

### 2.4. Measurements of Growth Performance

The amount of feed offered to each groups (Con and ProL) was the same. Specifically, feed was offered twice a day in feeders with suitably sized openings, reducing the possibility of the birds scavenging it in the litter. The amount of feed consumed by each group was determined once a week by weighing the amount of uneaten feed left in the feeders of each group. Based on the weight of the remaining and offered feed, the weekly average amount of feed consumed per day and the cumulative feed intake (CFI) were calculated.

The live body weight (LBW) for each bird in each group was determined on the day of placing the bird and then once a week, on days 7, 14, 21, 28, and 35. The average daily live weight gain (ADWG) of chickens and the FCR were determined using the formula provided by Chen et al. [6] in the following consecutive study periods: days 1–7, 8–14, 15–21, 22–28, and 29–35. Mortality was recorded once a day; the body weight of the bird and the cause of death were determined. It should be noted that chicken mortality during the study was lower than that under Latvia's production conditions (∼2%–5%).

### 2.5. Blood Sample Collection and Examination

According to our research methodology, and based on similar previous studies [10, 11], blood samples were collected from the wing vein of 45 randomly selected broilers of each group at the end of the study. The samples were examined in the laboratory of the Veterinary Clinic of Latvia University of Life Sciences and Technologies, using a Mindray BS200 Clinical Analyzer, with the absorbance photometry method. For this study, the following indicators were analysed: cholesterol (mmol/L), triglycerides (mmol/L), uric acid (mmol/L), total protein (g/L), albumin (g/L), and alkaline phosphatase (ALP, U/L).

### 2.6. Meat Sample Collection and Examination

To obtain a breast meat sample (without skin) that could reflect the production conditions, the broilers were stunned using the cervical dislocation method and bled. This procedure was carried out by a certified butcher; hence, the meat samples obtained during the study and the determined indicators are identical to meat that would be obtained under the production conditions. We obtained samples for meat quality examination from 30 randomly selected broilers of each group at the end of the study. After obtaining the samples, they were cooled, placed in polyethylene packaging intended for laboratory samples, and frozen at −22°C. Meat samples were investigated in the laboratory of the Scientific Institute of Food Safety, Animal Health, and Environment “BIOR” (Riga, Latvia). The following parameters were determined: cholesterol (mg/100 g), using method BIOR-T-012-132-2011, and PUFAs (g/100 g), *ω*-3 fatty acids (g/100 g), *ω*-6 fatty acids (g/100 g), *ω*-9 fatty acids (g/100 g), and SFAs (g/100 g), using method BIOR-T-012-131-2011. The PUFA/SFA and *ω*-6/*ω*-3 ratios were determined.

### 2.7. Statistical Analysis

We assessed the data distribution by using the Shapiro–Wilk test and by inspecting the normal Q-Q plots. We used Levene's test to test for homogeneity of variances. We determined the differences between the Con and ProL groups by using the independent samples *t*-test because the data were normally distributed and had homogenous variances. The results are presented as the mean ± standard deviation unless otherwise stated. We used Microsoft Excel to visualise the data and the Jamovi program [23] for statistical analysis. We considered *p*  <  0.05 to be a statistically significant difference.

## 3. Results and Discussion

### 3.1. Growth Performance

Since the ban on the prophylactic use of antibiotics, researchers have evaluated the potential of probiotics to improve growth performance in commercial poultry farms [24]. Probiotics improve chicken growth by participating in the processing of nutrients and by modulating the intestinal microflora and improving the barrier function of the intestinal walls. Probiotics lead to competitive exclusion of pathogenic bacteria as they compete with probiotics for nutrients, colonise the intestinal surface, leaving no attachment points for pathogenic microflora, and stimulate the immune system [25, 26].

The LBW, ADWG, CFI, and FCR did not differ significantly between the Con and ProL groups (*p*  >  0.05) (Table 2). To exclude the influence of the environment, pathogens and other stressors on the results, we tried to provide the optimal keeping conditions for the broilers of both groups throughout the study. Both groups performed extremely well in all phases of the study. We noted such high LBW and ADWG using lactic acid bacteria offered by the same manufacturer in only one study [22]; more often, LBW and ADWG were lower compared with the results of our study [6, 10, 27, 28]. However, when analysing the dynamics of the indicators, the curves of live weight and cumulative feed consumption initially increased slowly (from days 1 to 14), followed by a rapid increase in live weight and feed consumption (from days 15 to 35). This is the period when birds consume more food and grow faster. On the other hand, ADWG was initially higher (from days 1 to 28), mainly because, from their initial small starting weight, weight gain was faster; within a week, the chickens had doubled or even tripled their weight. The feed conversion curve increased gradually: as the birds grew larger, they consumed more feed. However, because the feed use efficiency was high, we did not observe a rapid increase in feed conversion.

Overall, there were very good production ratings in both study groups, and there were no significant differences between the groups. This could be explained by the fact that under favourable conditions, where the bird is not exposed to the risk of disease and stress; as in our study, the addition of probiotics to the feed may not give the expected results. This shows the significance and importance of microclimate and housing for broiler production and development [29, 30].

Similarly to our study, Sugiharto et al. [31] fed Lohmann broilers probiotics at different concentrations for 42 days and observed no significant changes in growth indicators (live weight, cumulative feed consumption, and the FCR), neither in individual study periods nor throughout the entire study period. Sarangi et al. [32] also found a similar dynamic in a 42-day study when they fed Vencobb broilers a probiotic mixture consisting of *Lactobacillus bulgaricus* and *Lactobacillus plantarum.* The authors reported a significantly higher live weight on day 14 of the study, but at the end of the study, there were no significant changes in live weight. In that study, the authors found no significant differences between the groups in indicators of cumulative feed consumption. They found a significantly lower FCR in the probiotic group between weeks 4 and 6 of the study, but in general, there were no significant changes between the groups [32].

Other studies have reported better results using probiotics. In their 21-day study, Agustono et al. [33] fed ISA brown male broilers a probiotic mixture consisting of *L. acidophilus*, *L. plantarum,* and *Bifidobacterium* spp. in different concentrations. They reported a significantly higher live weight in all groups that were fed probiotics. At the same time, there was significantly higher feed consumption; however, the FCR remained significantly lower in the study groups, indicating good feed use efficiency. Chen et al. [6] reported better results after feeding broilers (white leghorns) with *L. rhamnosus*. At the end of the study, the authors observed a significantly increased live weight and average daily gain and a significantly reduced FCR (*p*  <  0.05) compared with the group that was not fed probiotics. In several studies, the authors achieved significantly better results by feeding probiotics for 42 days. Palamidi et al. [3] fed male Cobb broilers a poultry-specific multispecies product consisting of five probiotic isolates (including *Limosilactobacillus reuteri* isolated from the hump) during the finisher period (days 29–42). Throughout the experimental period, the chickens achieved a significantly higher body weight gain and a significantly lower FCR, irrespective of the addition of live and inactivated forms of probiotics. Furthermore, in a 42-day study conducted by STI Biotechnologie [22], when broilers received a probiotic feed supplement containing *L. rhamnosus* and *L. farciminis* in different concentrations, and the authors observed a significantly reduced daily feed intake and a reduced FCR compared with groups that did not receive this supplement [22].

### 3.2. Blood Biochemical Indicators

Blood biochemical indicators provide extensive information on the animal's state of health. However, they can be affected by numerous factors: species, age, sex, housing conditions, nutrition, seasonal changes, and geographical region [34, 35]. To analyse serum blood biochemical indicators, we searched for scientifically confirmed data with defined normal physiological limits for broilers at the age of 30–40 days, but we could not find such data. Hence, we used the following studies with a neutral design and investigations that did not specify any other factors that influenced the results. Café [34] obtained blood biochemical data from broilers grown under thermoneutral conditions. Angoua Kokore et al. [35] compared blood biochemical indicators of local and meat broiler breeds. Shuvo et al. [36] evaluated the effect of 35 days of *Lactobacillus* supplement feeding on broiler chickens, including blood parameters.

We found that almost all parameters (Table 3) did not differ significantly (*p*  >  0.05) between the Con and ProL groups, except for cholesterol (*p*=0.039), with a difference of 0.26 mmol/L (95% confidence interval (CI) 0.01–0.51, *d* = 0.45 (medium effect size)). The cholesterol level in the study by Café [34] was 3.25 mmol/L, which is 7.14% lower than that in the ProL group of our study and 13.56% lower than that in the Con group. In the study by Shuvo et al. [36], the average cholesterol level in the control group was only 2.74 ± 0.06 mmol/L, but in the study group, it was even lower (2.12 ± 0.12 mmol/L). The authors explained this outcome by referring to the presence of lactic acid bacteria in the fermented feed and the ability of these bacteria to collect and bind cholesterol. As a result, the total cholesterol level in the blood serum would decrease due to the inhibition of bile acid absorption in the intestine [36, 37]. Mohan et al. [38] also explained the ability of acidophilic bacteria to decrease cholesterol levels in the blood by reducing its absorption and/or synthesis in the intestines.

Cholesterol is an important component of cell membranes: it ensures cell elasticity and membrane permeability and participates in the synthesis of important hormones (e.g., sex hormones and hormones of the adrenal cortex) and bile acid; a small amount of it is also needed for the synthesis of vitamin D [39]. Although we found a significantly lower blood cholesterol level in the ProL group, it is still higher than the levels reported in previous studies. We would like to add that there is no reason to consider the blood cholesterol level we found to be elevated (above the physiological norm) because the animals in our study were in good health, as evidenced by the high productivity rates.

Similarly to our research, Hashemzadeh et al. [40] fed male Ross 308 broiler chicks *L. rhamnosus* for 42 days and reported a significantly lower blood cholesterol level. Ghorbani et al. [41] also achieved similar results in a 42-day study by feeding the commercial probiotic “Primalac” to Ross 308 broilers; at the end of the study, the blood cholesterol level was significantly lower than that in the control group. Yazhini et al. [42] also reported positive results in a 42-day study in which they fed broiler chicks nonencapsulated and encapsulated *Lactococcus lactis* and *Bifidobacterium bifidum*; in all probiotic groups, the authors observed a significantly lower total cholesterol level. In addition, Hussein and Selim [5], fed broilers probiotics, including *L. acidophilus* and found that the total blood cholesterol level decreased significantly compared with the group that did not receive probiotics. However, Zhu et al. [8] described opposite results. Female yellow-feathered broilers fed heat-inactivated compound probiotic in a 63-day study showed a significantly reduced blood cholesterol level on day 42 of the study, but no significant difference on day 63. Sugiharto et al. [43] also reported that there was not a significant reduction in the blood cholesterol levels in any of the study groups of Indonesian indigenous crossbred chicks in a 10-week study by feeding *B. subtilis* and a multistrain probiotic preparation in combination with vitamins and minerals.

Although we did not find significant differences in the other blood biochemical indicators, we highlight certain indicators (triglycerides, total protein, and albumin).

The blood triglyceride concentration was 6.95% higher in the ProL group (0.72 ± 0.31 mmol/L) compared with the Con group (0.67 ± 0.30 mmol/L). In the study by Café [34], the triglyceride concentration was even higher (0.84 mmol/L) on day 35. Triglycerides are some of the most important plasma lipids and are either ingested with food (exogenous triglycerides) or synthesised in the liver (endogenous triglycerides). They are stored in adipose tissue, and only a small amount circulates in the blood. If the levels of fatty acids and carbohydrates in the blood are too high, they are converted and stored in the body as triglycerides [39]. Therefore, in our study, given the same feed ratio, the chickens in the ProL group digested the feed more successfully, providing a higher level of triglyceride raw material.

The level of total protein in the blood was lower in the chickens of the ProL group (33.03 ± 6.49 g/L) compared with the Con group (34.92 ± 7.26 g/L). Café [34] reported a total protein level of 25–45 g/L in blood, but it was higher in the study by Angoua Kokore et al. [35], specifically 52.2 ± 6.4 g/L. However, the albumin levels in our study were higher in the ProL group (15.45 ± 2.79 g/L) than those in the Con group (15.03 ± 3.01 g/L). Café [34] reported lower albumin levels than either group of our study (12.7 g/L), but Shuvo et al. [36] reported a higher level for the control group (17.8 ± 1.9 g/L). Albumin is a plasma protein that is synthesised in the liver for ingested and absorbed nutrients. It constitutes more than half of the total protein (the second largest fraction is represented by globulins). Albumin maintains colloidal osmotic pressure and acts as a carrier protein for hormones, fatty acids, bilirubin, calcium ions, and drugs, among others. Albumin levels can indicate liver function and protein metabolism in the body [39]. Although there was not a significant difference between the ProL and Con groups, a higher level of albumin in the blood indicates more successful liver function and protein exchange in the body.

Other authors have also studied the ability of lactic acid bacteria to positively influence these blood biochemical indicators in birds. Hosseini et al. [9] fed male Cobb broiler chicks the commercial probiotic mix Protexin, which contains several *Lactobacillus* spp., including *L. rhamnosus*, and reported significantly higher concentrations of total protein and albumin as well as a significantly lower blood triglyceride concentrations compared with the control group and the group to which antibiotics were added to the diet. Hussein and Selim [5] reported similar results: a significantly higher concentration of total protein, including albumin and globulin, as well as a significantly reduced concentration of total lipids in the blood. In a 35-day study in which broilers were fed different concentrations of a probiotic containing *L. casei*, Astuti et al. [10], achieved significantly higher plasma total protein levels in all probiotic groups. However, Qorbanpour et al. [7], Shah et al. [11], and Zhu et al. [8] fed broilers with probiotics, which included *Lactobacillus* spp. and did not observe significant differences at the end of the study in the blood biochemical parameters compared with the control group. In contrast, Sugiharto et al. [43] found significantly lower blood triglyceride and total protein (including albumins and globulins) concentrations (*p*  <  0.05), but no significant differences in the ratio of albumins to globulins.

### 3.3. Meat Indices

In the obtained breast meat samples, the ProL group showed significantly higher cholesterol and *ω*-6 fatty acid levels, namely, 12.92 ± 0.87 mg/100 g and 29.32 ± 1.58 g/100 g (*p*  <  0.05), respectively, whereas the Con group showed significantly higher *ω*-9 fatty acid and SFA levels, with 40.33 ± 1.82 g/100 g and 29.01 ± 2.5 g/100 g (*p*  <  0.05), respectively. Based on Cohen's *d*, the recorded differences for all parameters have a high effect size (Table 4).

Because feeding a mixture of *L. farciminis* and *L. rhamnosus* for 35 days significantly reduced the blood cholesterol levels, we expected that its level in breast meat samples would also be lower in the ProL group. However, the breast cholesterol level was significantly higher (*p*  <  0.05) than that in the Con group. According to published studies, the cholesterol level in breast muscle without skin ranges from 77 to 85 mg/100 g [44, 45], but in the breast meat samples (also without skin) submitted to our laboratory, it was 12.92 ± 0.87 mg/100 g (ProL) and 10.02 ± 0.35 mg/100 g (Con)–7–8 times lower. According to the findings of Conchillo et al. [44], freezing the meat samples before examination does not affect their cholesterol level. Therefore, by using age-appropriate feed in chicken breeding, as well as providing the best possible keeping conditions and regulating the light/dark regime, an especially low cholesterol level can be achieved in breast muscle meat (10–13 mg/100 g of product), as shown by the results of our study.

Chicken breast muscle is a valuable source of essential fatty acids, especially *ω*-3 LC-PUFAs [46]. *ω*-3 and *ω*-6 LC-PUFAs have been studied to help prevent and improve the outcome of chronic diseases in humans, including metabolic, cardiovascular, and neurodegenerative diseases as well as certain types of cancer [18, 46, 47]. In our study, the *ω*-3 fatty acid level was 16.25% higher in the ProL group than in the Con group, although this difference was not significant (*p*=0.104). However, the *ω*-6 fatty acid level was significantly higher in the breast muscle of the ProL group (*p*=0.001). In this sense, the addition of *L. farciminis* and *L. rhamnosus* to the feed of broilers increased both *ω*-3 and *ω*-6 fatty acids in breast meat, without using other feed additives containing, these fatty acids (fish or other oils) and without producing an unpleasant taste and smell [18]. It is important to note that the intake of *ω*-3 and *ω*-6 fatty acids is important to maintain human health. In recent years, concerns regarding the potential role of *ω*-6 fatty acids in inflammation, thrombosis, and low-density lipoprotein oxidation have been dismissed, and it is recommended that at least 5%–10% of the daily energy intake should come directly from *ω*-6 fatty acids [48].

Researchers are also interested in the *ω*-6/*ω*-3 ratio in the human diet, as deviations contribute to the pathogenesis of cardiovascular disease, cancer, inflammation, and many autoimmune diseases [18, 47]. As food availability and eating habits change, the *ω*-6/*ω*-3 ratio in human diets today ranges from 10/1 to 20/1, in contrast to the 1/1 ratio found in our ancestors' diets [49]. According to our results, this ratio was 8.54 ± 2.03 in the ProL group and 8.64 ± 2.41 in the Con group, which is less (and therefore healthier) than that in the modern human diet [49].

After 35 days of consuming a feed supplement containing lactic acid bacteria, SFAs also decreased significantly in the breast meat samples of the ProL group compared with the Con group (*p*=0.031). Given that SFAs are less healthy than PUFAs [47], reducing their amount is a significant achievement. The levels of fatty acids, especially LC-PUFAs, in chicken meat can be modified more easily than those in other livestock meats [50].

According to the recommendations of the UK Ministry of Health [51] and other authors, it is important to consider the PUFA/SFA ratio [51, 52]. Under our housing and feeding conditions, the inclusion of probiotics in the feed of broilers did not significantly change the PUFA/SFA ratio: it was 2.68 ± 0.24 for the ProL group and 2.48 ± 0.25 for the Con group. Although the inclusion of a mixture of lactic acid bacteria did not significantly affect this ratio, pathogen-free conditions ensured very good quality indicators, which encourage further research in this direction.

## 4. Conclusions

The probiotic mixture containing *L. farciminis* and *L. rhamnosus* added to feed in a 35-day study did not significantly affect the growth (LBW and ADWG) and feed intake indicators (CFI and FCR) of specific pathogen-free broilers. Feeding these probiotics significantly reduced the blood cholesterol level but did not produce the expected reduction in meat cholesterol. However, probiotic feeding increased the amount of PUFAs (*ω*-3 and *ω*-6) in the breast meat of broilers and decreased SFAs. The main novelty of the study is that we evaluated the effects of probiotics for specific pathogen-free Ross 308 broilers. The combination of *L. farciminis* and *L. rhamnosus* affected broiler growth, meat quality, and some blood biochemical indicators. These changes mean that the chicks did not have to deal with additional stress factors during intense growth, which proves the direct impact of these bacteria on the chicks. The positive results, albeit contradictory, show that it is necessary to continue to search for the most suitable combinations, doses, and feeding duration of probiotics in poultry farming for each breed and age. In future studies, histological and immunohistochemical examinations should be performed to evaluate in more detail the effect of the combination of lactic acid bacteria (*L. farciminis* and *L. rhamnosus*) on the growth and development of broiler chickens.

## Figures and Tables

**Table 1:** Analytical composition and physiological additives of the basal diet at each feeding stage.

Components	Starter diet	Grower diet	Finisher diet
*Crude*			
Protein, %	22.50	21.50	19.50
Fibre, %	2.40	2.86	2.83
Fat, %	4.24	5.20	7.22
Ash, %	4.32	4.73	3.68
*Essential amino acids*			
Lysine, %	1.36	1.20	1.14
Methionine, %	0.84	0.60	0.85
*Minerals*			
Ca, %	0.96	1.00	0.78
Na, %	0.35	0.16	0.19
P, %	0.50	0.50	0.50
*Vitamins*			
A, SV/kg	16,900	14,300	13,000
D_3_, SV/kg	6,500	5,500	5,000
E, mg/kg	104,0	88.0	80.0
*Micronutrients*			
FeSO_4_, mg/kg	22.1	18.7	17.0
Ca (IO_3_)_2_, mg/kg	1.63	1.38	1.25
CuSO_4_, mg/kg	20.8	17.6	16.0
MnO₂, mg/kg	156.0	132.0	120.0
ZnO, mg/kg	117.0	99.0	90.0
Na_2_SeO_3_, mg/kg	0.39	0.33	0.30

**Table 2:** Mean and 95% confidence interval of the live body weight, average daily weight gain, cumulative feed intake, and feed conversion ratio of broilers until day 35.

Parameters	Periods (day)	Control group mean (95% confidence interval)	Probiotic group mean (95% confidence interval)	*p*
Live body weight (g)	0	43.8 (42.1–45.5)	44.5 (42.5–46.4)	0.945
	7	229.2 (225.6–232.8)	216.7 (216.4–223.1)	0.831
	14	582.2 (572.8–591.5)	588.6 (578.9–598.1)	0.834
	21	1154.1 (1136.7–1171.5)	1166.2 (1147.4–1184.9)	0.979
	28	1957.7 (1931.0–1984.4)	1961.9 (1935.0–1988.9)	0.989
	35	2828.0 (2784.2–2871.8)	2835.7 (2791.7–2879.7)	0.947

Average daily weight gain (g)	1–7	23.8 (17.7–29.9)	23.5 (18.9–28.0)	0.867
	8–14	53.0 (43.9–62.0)	54.1 (41.6–66.6)	0.770
	15–21	83.7 (66.4–101.1)	85.1 (62.0–108.3)	0.845
	22–28	112.7 (90.6–134.8)	111.0 (87.3–134.7)	0.833
	29–35	124.3 (110.6–138.0)	124.8 (99.6–149.9)	0.947

Cumulative feed intake (g)	1–7	219.2 (206.3–232.1)	218.3 (205.9–230.6)	0.829
	8–14	640.2 (600.8–679.7)	639.1 (592.3–681.9)	0.832
	15–21	1323.6 (1225.5–1421.7)	1322.8 (1234.6–1410.9)	0.979
	22–28	2344.9 (2284.7–2405.1)	2344.9 (2263.2–2426.6)	0.999
	29–35	3620.3 (3456.2–3784.5)	3601.9 (3346.1–3857.8)	0.807

Feed conversion ratio	1–7	1.0 (0.8–1.3)	1.0 (0.8–1.2)	0.998
	8–14	1.1 (0.9–1.3)	1.1 (0.9–1.3)	0.794
	15–21	1.1 (0.9–1.3)	1.1 (0.9–1.3)	0.835
	22–28	1.2 (1.1–1.3)	1.2 (1.1–1.3)	0.944
	29–35	1.3 (1.2–1.4)	1.3 (1.2–1.4)	0.757

**Table 3:** Differences in the biochemical parameters of broiler blood on day 35 between the control and probiotic group.

Parameters	Control			Probiotic			*p*	*d*
	M	SD	SE	M	SD	SE		
Cholesterol (mmol/L)	3.76	0.57	0.09	3.50	0.59	0.09	0.039	0.45
Triglycerides (mmol/L)	0.67	0.30	0.04	0.72	0.31	0.05	0.505	NA
Urea (mmol/L)	0.34	0.15	0.02	0.33	0.16	0.03	0.921	NA
Total protein (g/L)	34.92	7.26	1.11	33.03	6.49	1.00	0.210	NA
Albumin (g/L)	15.03	3.01	0.46	15.45	2.79	0.43	0.508	NA
Alkaline phosphatase (U/L)	2,917.95	1,487.77	226.88	2,725.16	1,545.43	238.47	0.559	NA

*Notes*. SE: standard error; *d*: Cohen's effect size; M: arithmetic mean; NA: not applicable; SD: standard deviation.

**Table 4:** Differences in meat quality parameters of 35-day-old broilers between the control and probiotic groups.

Parameters	*Control*			*Probiotic*			*p*	*d*
	M	SD	SE	M	SD	SE		
Cholesterol, mg/100 g	10.02	0.35	0.11	12.92	0.87	0.28	0.001	4.35
PUFAs, g/100 g	71.66	4.26	1.35	71.39	2.70	0.85	0.867	NA
Omega-3, g/100 g	2.99	0.82	026	3.57	0.65	0.21	0.104	NA
Omega-6, g/100 g	24.20	1.23	0.39	29.32	1.58	0.50	0.001	3.62
Omega-9, g/100 g	40.33	1.82	0.58	37.51	1.07	0.34	0.001	1.89
SFAs, g/100 g	29.01	2.50	0.79	26.70	1.88	0.59	0.031	1.05
Omega-6/omega-3	8.64	2.41	0.77	8.54	2.03	0.64	0.921	NA
PUFAs/SFAs	2.48	0.25	0.08	2.68	0.24	0.08	0.827	NA

*Notes*. *d*: Cohen's *d* effect size; NA: not applicable; SE: standard error; M: arithmetic mean; PUFAs: polyunsaturated fatty acids; SD: standard deviation; SFA: saturated fatty acids.

## Data Availability

The data used to support the conclusions of this study are available from the corresponding author upon reasonable request.
